# Minimally invasive robot-assisted and laparoscopic distal pancreatectomy in a pan-European registry a retrospective cohort study

**DOI:** 10.1097/JS9.0000000000001315

**Published:** 2024-03-18

**Authors:** Eduard A. van Bodegraven, Tess M. E. van Ramshorst, Svein O. Bratlie, Arto Kokkola, Ernesto Sparrelid, Bergthor Björnsson, Dyre Kleive, Stefan K. Burgdorf, Safi Dokmak, Bas Groot Koerkamp, Santiago Sánchez Cabús, I Quintus Molenaar, Ugo Boggi, Olivier R. Busch, Miha Petrič, Geert Roeyen, Thilo Hackert, Daan J. Lips, Mathieu D’Hondt, Mariëlle M E Coolsen, Giovanni Ferrari, Bobby Tingstedt, Alejandro Serrablo, Sebastien Gaujoux, Marco Ramera, Igor Khatkov, Fabio Ausania, Regis Souche, Sebastiaan Festen, Frederik Berrevoet, Tobias Keck, Robert P. Sutcliffe, Elizabeth Pando, Roeland F. de Wilde, Beatrice Aussilhou, Paul S. Krohn, Bjørn Edwin, Per Sandström, Stefan Gilg, Hanna Seppänen, Caroline Vilhav, Mohammad Abu Hilal, Marc G. Besselink

**Affiliations:** a Department of Surgery, Amsterdam UMC, University of Amsterdam, Amsterdam, The Netherlands; b Cancer Center Amsterdam, The Netherlands; c Department of General Surgery, Istituto Ospedaliero Fondazione Poliambulanza, Brescia, Italy; d Department of Surgery, Sahlgrenska University Hospital, Gothenburg, Sweden; e Department of Surgery, University of Helsinki, Helsinki University Hospital, Helsinki, Finland; f Division of Surgery, Department of Clinical Science, Intervention and Technology, Karolinska Institutet, Karolinska University Hospital, Stockholm, Sweden; g Department of Surgery in Linköping and Department of Biomedical and Clinical Sciences, Linköping University, Linköping, Sweden; h Department of HPB Surgery, The Intervention Centre, Oslo University Hospital and Institute for Clinical Medicine, Oslo, Norway; i Department of Surgery and Transplantation, Rigshospitalet Copenhagen University Hospital, Copenhagen, Denmark; j Departement of HPB Surgery and Liver Transplantation, APHP Beaujon Hospital, University of Paris Cité, Clichy, France; k Department of Surgery, Erasmus MC Cancer Institute, Rotterdam, The Netherlands; l Department of HPB Surgery, Hospital de la Santa Creu I Sant Pau, Barcelona, Spain; m Department of Surgery, Regional Academic Cancer Centre Utrecht, UMC Utrecht Cancer Centre and St Antonius Hospital Nieuwegein, University Medical Centre Utrecht, Utrecht, The Netherlands; n Division of General and Transplant Surgery, University of Pisa, Pisa, Italy; o Department of Abdominal Surgery, Ljubljana University Medical Center, Zaloška cesta, Ljubljana, Slovenia; p Department of HPB, Endocrine and Transplantation Surgery, University Hospital Antwerp, Edegem, Belgium and University of Antwerp, Wilrijk, Belgium; q Department of Surgery, Heidelberg University Hospital, Heidelberg, Germany; r Department of Surgery, Medisch Spectrum Twente, Enschede, The Netherland; s Department of Digestive and Hepatobiliary/Pancreatic Surgery, Groeninge Hospital, Kortrijk, Belgium; t Department of Surgery, Maastricht University Medical Center+, University of Maastricht, Maastricht, The Netherlands; u Division of Minimally Invasive Surgical Oncology, ASST Grande Ospedale Metropolitano Niguarda, Milan, Italy; v Department of Surgery, Clinical Sciences Lund, Lund University, Skåne University Hospital, Lund, Sweden; w HPB Surgical Division, Miguel Servet University Hospital, Zaragoza, Spain; x Department of Hepatobiliary and Pancreatic Surgery and Liver Transplantation, AP-HP, Pitié-Salpêtrière Hospital, Sorbonne University, Paris, France; y Department of Clinical and Experimental Sciences, University of Brescia, Brescia, Italy; z Department of Surgery, Moscow Clinical Scientific Center, Moscow, Russia; aa Department of HPB and Transplant Surgery, Hospital Clinic, IDIBAPS, University of Barcelona, Barcelona, Spain; ab Department of Surgery, Saint-Éloi Hospital, Montpellier, France; ac Department of Surgery, OLVG, Amsterdam, The Netherlands; ad Department of General and HPB Surgery and Liver Transplantation, Ghent University Hospital, Ghent, Belgium; ae Department of Surgery, University Medical Centre Schleswig-Holstein, Campus Lübeck, Lübeck, Germany; af Department of Hepatopancreatobiliary Surgery, University Hospitals Birmingham NHS Foundation Trust, Birmingham, UK; ag Department of Hepato, Pancreato, Biliary, and Transplant Surgery, Hospital Universitari Vall d’Hebron, Barcelona, Spain

**Keywords:** E-MIPS registry, laparoscopic distal pancreatectomy, minimally invasive distal pancreatectomy, registry-based outcome, robot-assisted distal pancreatectomy

## Abstract

**Background:**

International guidelines recommend monitoring the use and outcome of minimally invasive pancreatic surgery (MIPS). However, data from prospective international audits on minimally invasive distal pancreatectomy (MIDP) are lacking. This study examined the use and outcome of robot-assisted (RDP) and laparoscopic (LDP) distal pancreatectomy in the E-MIPS registry.

**Patients and methods:**

Post-hoc analysis in a prospective audit on MIPS, including consecutive patients undergoing MIDP in 83 centers from 19 European countries (01-01-2019/31-12-2021). Primary outcomes included intraoperative events (grade 1: excessive blood loss, grade 2: conversion/change in operation, grade 3: intraoperative death), major morbidity, and in-hospital/30-day mortality. Multivariable logistic regression analyses identified high-risk groups for intraoperative events. RDP and LDP were compared in the total cohort and high-risk groups.

**Results:**

Overall, 1672 patients undergoing MIDP were included; 606 (36.2%) RDP and 1066 (63.8%) LDP. The annual use of RDP increased from 30.5% to 42.6% (*P*<0.001). RDP was associated with fewer grade 2 intraoperative events compared with LDP (9.6% vs. 16.8%, *P*<0.001), with longer operating time (238 vs. 201 min, *P*<0.001). No significant differences were observed between RDP and LDP regarding major morbidity (23.4% vs. 25.9%, *P*=0.264) and in-hospital/30-day mortality (0.3% vs. 0.8%, *P*=0.344). Three high-risk groups were identified; BMI greater than 25 kg/m^2^, previous abdominal surgery, and vascular involvement. In each group, RDP was associated with fewer conversions and longer operative times.

**Conclusion:**

This European registry-based study demonstrated favorable outcomes for MIDP, with mortality rates below 1%. LDP remains the predominant approach, whereas the use of RDP is increasing. RDP was associated with fewer conversions and longer operative time, including in high-risk subgroups. Future randomized trials should confirm these findings and assess cost differences.

## Introduction

Highlights

- In the first three years of the E-MIPS registry, minimally invasive distal pancreatectomy is mostly performed laparoscopically, although the robotic approach is used increasingly.
- Robot-assisted and laparoscopic distal pancreatectomy are both safe and appropriate alternatives.
- People with a high BMI, previous abdominal surgery, and vascular involvement are at risk for intraoperative events in minimally invasive distal pancreatectomy.
- Robot-assisted distal pancreatectomy is associated with lower conversion rates and longer operative time, including in high-risk subgroups.

Minimally invasive pancreatic surgery (MIPS) is increasingly being adopted worldwide but remains associated with a high risk of postoperative morbidity^1^. Therefore, both the Brescia European Guidelines for Minimally Invasive Pancreatic Surgery (EGUMIPS)^2^ and Miami Guidelines^3^ strongly encourage national and international registries to monitor the use and outcome of MIPS. In 2019, a pan-European registry was founded by the European Consortium on Minimally Invasive Pancreatic Surgery (E-MIPS). The E-MIPS registry collects data on minimally invasive pancreatic resections, including minimally invasive distal pancreatectomy (MIDP) and pancreatoduodenectomy to improve outcomes through research, training, and quality control.

Currently, the E-MIPS registry includes over 100 participating centers from 27 countries. The registry includes both robot-assisted distal pancreatectomy (RDP) and laparoscopic distal pancreatectomy (LDP). Since the safety and efficacy of MIDP have extensively been proven in previous literature^4–6^, interest in RDP is growing. Potential benefits of RDP include increased instrument dexterity, vision, and surgeon ergonomics, potentially leading to lower conversion rates^7^.

Recent systematic reviews comparing RDP with LDP have reported favorable outcomes of RDP but were mainly based on retrospective, single-center studies^8–10^ as randomized trials directly comparing RDP and LDP are lacking. In addition, despite retrospective studies comparing both approaches, studies investigating subgroups or patients who would benefit the most from a particular approach are lacking. In the current study, data from the first 3 years of the prospectively maintained E-MIPS registry was used to provide an overview of MIDP across Europe and to compare the use and outcome of RDP and LDP.

## Methods

Post-hoc analysis of all consecutive patients undergoing MIDP from the prospectively maintained E-MIPS registry, between the inception of the registry on 1 January 2019 up and 31 December 2021. All procedures were registered in an online-secured GCP-certified data storage system (CASTOR, CIWIT B.V., Amsterdam). At each participating center, a local study coordinator was appointed who received login credentials to enter the data in the online-secured database comprising all parameters of interest, including definitions. Three centers per year were randomly allocated and audited by the E-MIPS registry coordinators to perform a data quality check. This study was conducted according to the principles of the Declaration of Helsinki (64th version, October 2013), by the Medical Research Involving Human Subjects Act (WMO), besides other guidelines, regulations, and acts. Ethical approval was waived due to the observational nature of the study. All aspects of the project were handled by the Strengthening The Reporting Of Cohort Studies in Surgery (STROCSS)^11^ guidelines. Supplemental Digital Content 1, http://links.lww.com/JS9/C136.

### Definitions

Preoperative variables included baseline and tumor characteristics such as sex, age, American Society of Anesthesiologists (ASA) classification^12^, body mass index (BMI), previous abdominal surgery, preoperative diagnosis, and vascular involvement other than splenic artery/vein (i.e. portal vein, superior mesenteric vein/artery, celiac axis, and/or hepatic artery). Intraoperative events were classified according to the modified Satava classification: grade 1; excessive blood loss (not requiring conversion), grade 2; conversion to laparotomy or major change in operation, grade 3: intraoperative death^13,14^. Conversion as a separate variable was defined as an attempted minimally invasive resection that required conversion to laparotomy or hand assistance for reasons other than trocar placement or specimen extraction^15^. Data on postoperative outcomes were recorded up to 30 days postoperatively. Postoperative complications were classified using the Clavien-Dindo classification of surgical complications, major morbidity was defined as Clavien-Dindo 3a or higher^16^. Only grade B/C pancreatic-specific complications were included, i.e. postoperative pancreatic fistula (POPF), delayed gastric emptying (DGE), and postpancreatectomy hemorrhage (PPH), following the definitions of the International Study Group for Pancreatic Surgery (ISGPS)^17–19^. Resection margins were categorized according to the Royal College of Pathologists definition and classified into R0 (distance margin to tumor ≥ 1 mm), R1 (distance margin to tumor<1 mm), and R2 (macroscopically positive margin)^20^.

### Outcome measures

Primary outcomes focused on intraoperative events based on the modified Satava classification^13,14^, major morbidity, and in-hospital/30-day mortality (i.e. mortality during the entire hospital stay, also beyond 30 d, and in case of earlier discharge mortality until 30 d postoperatively). Secondary outcomes included intraoperative variables such as operation time, blood loss, and conversion, postoperative variables such as grade B/C POPF^17^, DGE^18^, and PPH^19^, reoperation, readmissions and length of hospital stay, and oncological variables as histopathological tumor type, lymph node yield, margin status, and tumor size.

### Statistical analysis

Data were analyzed using IBM SPSS Statistics for Windows version 26.0 (IBM Corp., Orchard Road Armonk, New York, US). Data analyses were performed according to the intention-to-treat principle (i.e. converted procedures were included in the minimally invasive group) and performed by the study coordinators EAVB and TVR, where after crosschecked by a dedicated statistician from Amsterdam UMC. Categorical data were presented as proportions and continuous data as mean with standard deviations (SD) in case of normally distributed data, or median with interquartile range (IQR) in case of non-normally distributed data. Student t, Mann–Whitney *U*, Kruskal–Wallis, *χ*^2^, or Fisher’s exact tests were used as appropriate. Univariable and multivariable logistic regression analyses were performed to identify variables associated with intraoperative Satava events (endpoint). High-risk groups were defined based on significant variables in multivariable analysis. Comparative analyses were performed between RDP and LDP in the total cohort and the high-risk groups. An unplanned sensitivity analysis was performed excluding patients with previous abdominal surgery. A flowchart of the study methodology and analyses is shown in Supplementary Figure 1, Supplemental Digital Content 2, http://links.lww.com/JS9/C137. Variables with a *P* value less than 0.20 in univariable analysis or potentially associated with a particular approach based on the literature were considered for multivariable analysis. Multivariable analysis was performed using backward selection with a *P* value of less than 0.10, presented as odds ratios (OR) with corresponding 95% confidence intervals (CI). A *P* value of less than 0.05 was considered to indicate statistical significance.

## Results

### Patient and center demographics

During the study period, 1672 patients after MIDP were included from 83 centers in 19 countries. This entailed 606 (36.2%) patients after RDP and 1066 (63.8%) patients after LDP. In 2019, 557 MIDPs were performed in 60 centers, in 2020, 509 MIDPs in 61 centers, and in 2021, 606 MIDPs in 63 centers. The total number of inclusions per center during the study period is shown in Supplementary Figure 2, Supplemental Digital Content 2, http://links.lww.com/JS9/C137.

Among the 83 participating centers, 16 centers (19.3%) performed only RDP (*n*=244), 40 centers (48.2%) only LDP (*n*=687), and 27 centers (32.5%) performed both (*n*=741). The baseline and tumor characteristics of all MIDPs are shown in Table 1. Pancreatic ductal adenocarcinoma (PDAC) and pancreatic neuroendocrine tumor (pNET) were the most common indications for MIDP (*n*=422, 28.4% and *n*=417, 28.0%, respectively). Baseline characteristics were comparable between RDP and LDP regarding age, sex, ASA greater than or equal to III, BMI greater than 25 kg/m^2^, tumor size, and vascular involvement. Patients in the RDP group had less previous abdominal surgery (25.5% vs. 32.9%, *P=*0.003).

**Table 1:** Baseline characteristics for patients undergoing minimally invasive distal pancreatectomy.

	Total MIDP (n=1672)	RDP (n=606)	LDP (n=1066)	*P*
Age, years, median, (IQR)	66 (54 - 74)	66 (55–75)	65 (53–73)	0.050
Female, sex, n, (%)	922 (55.1)	328 (54.1)	594 (55.7)	0.528
BMI, kg/m^2^, median, (IQR)	25.9 (23.1–29.4)	25.6 (22.6–28.9)	26.2 (23.4–29.5)	**0.003**
BMI>25 kg/m^2^, n, (%)	985 (59.5)	340 (56.9)	645 (61.0)	0.102
ASA, n (%)				—
1	240 (14.7)	52 (8.8)	188 (18.0)	
2	890 (54.4)	341 (57.9)	549 (52.4)	
3	487 (29.8)	187 (31.7)	300 (28.7)	
4	19 (1.2)	9 (1.5)	10 (1.0)	
ASA ≥ III, n, (%)	506 (30.9)	196 (33.3)	310 (29.6)	0.123
Previous abdominal surgery, n, (%)	472 (30.4)	140 (25.5)	332 (32.9)	**0.003**
Vascular involvement, n, (%)	49 (3.0)	17 (2.9)	32 (3.1)	0.786
Tumor size, mm, median (IQR)	28.0 (17.0 – 44.0)	28.0 (18.0 – 42.0)	28.4 (17.0 – 45.0)	0.892
Tumor size>50 mm, n, (%)	245 (16.2)	78 (14.7)	167 (17.0)	0.240
Preoperative diagnosis, n, (%)				—
PDAC	422 (28.4)	158 (30.0)	264 (27.5)	
pNET	417 (28.0)	134 (25.4)	283 (29.5)	
IPMN	315 (21.2)	122 (23.1)	193 (20.1)	
Cystic lesion	248 (16.7)	84 (15.9)	164 (17.1)	
SPN	49 (3.3)	20 (3.8)	29 (3.0)	
Chronic pancreatitis	20 (1.3)	7 (1.3)	13 (1.4)	

Values in parentheses are percentages unless mentioned otherwise. Percentages may not add up due to rounding and missing data.

*P* values report on the statistical difference between RDP and LDP.

ASA, American Society of Anesthesiologists; BMI, body mass index; IPMN, intraductal papillary mucinous neoplasm; PDAC, pancreatic ductal adenocarcinoma; pNET, pancreatic neuroendocrine tumor; SD, standard deviation; SPN, solid pseudopapillary neoplasm.

Statistical significance (P<0.05) values are in bold.

### Time trends

**Figure 1:**
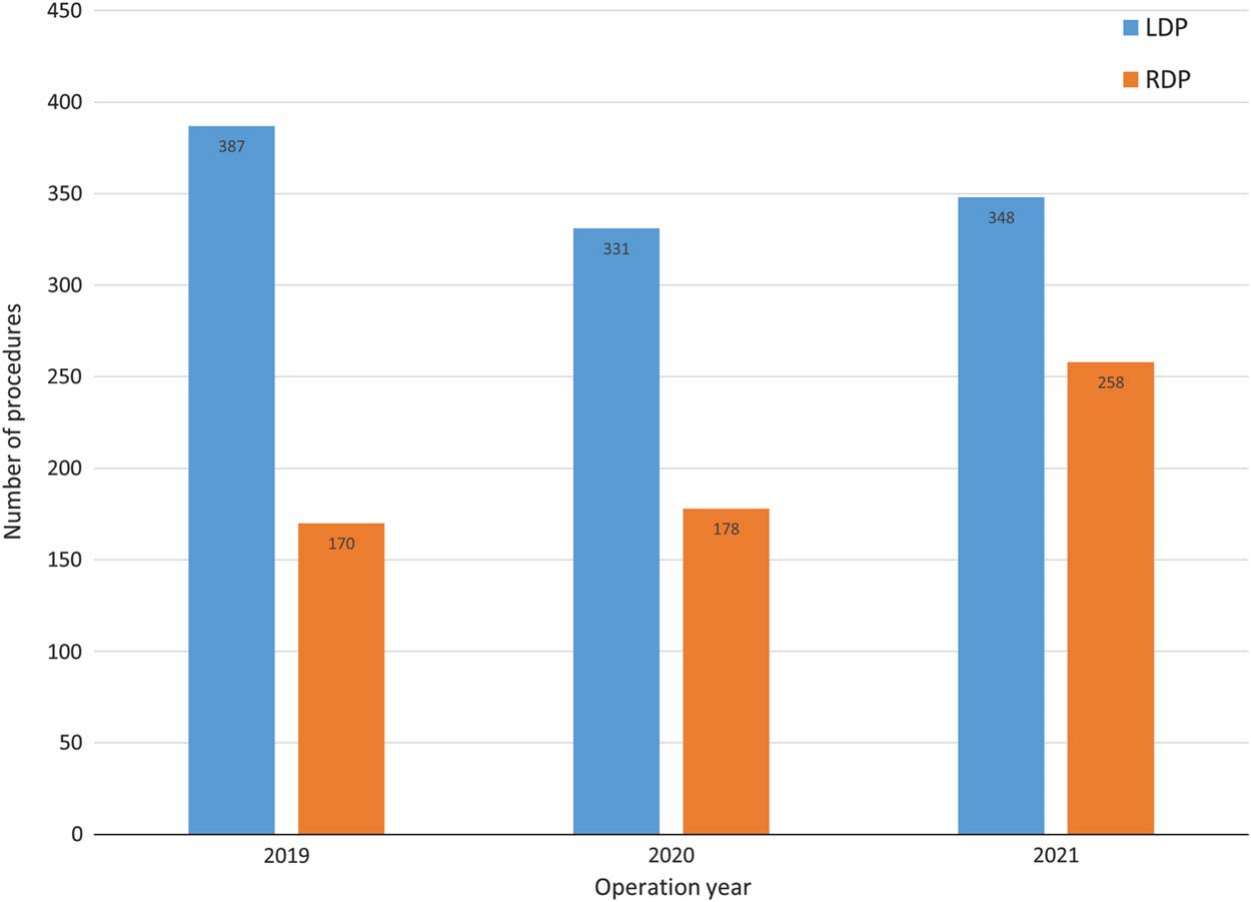
Use of robot-assisted (RDP) and laparoscopic (LDP) distal pancreatectomy in the period 2019-2021.

Among patients undergoing MIDP, the rate of BMI greater than or equal to 25 kg/m^2^ (56.1%, 56.6%, 65.0%, *P=*0.003) and ASA greater than or equal to III (25.7%, 30.2%, 36.6%, *P*<0.001) increased over time (Table 2). Also, more patients were operated on for a malignant indication (PDAC), despite a decrease in 2021 (23.5%, 35.0%, 26.9%, *P*<0.001). No differences were observed in patient age, previous abdominal surgery, tumor size greater than 50 mm, and vascular involvement over time (Table 2). The use of RDP among all patients undergoing MIDP increased (30.5%, 35.0%, 42.6%, *P*<0.001), as shown in Fig. 1.

**Table 2:** Patient selection for minimally invasive distal pancreatectomy, 2019–2021.

	2019 (n=557)	2020 (n=509)	2021 (n=606)	*P*
Age ≥ 65 y	298 (53.5)	269 (52.8)	317 (52.5)	0.941
Female, n, (%)	332 (59.6)	277 (54.4)	313 (51.7)	**0.023**
BMI ≥25 kg/m^2^, n, (%)	309 (56.1)	286 (56.6)	390 (65.0)	**0.003**
ASA ≥ III, n, (%)	142 (25.7)	152 (30.2)	212 (36.6)	**<0.001**
Previous abdominal surgery, n, (%)	168 (30.2)	137 (34.4)	167 (27.9)	0.091
Vascular involvement, n, (%)	13 (2.4)	18 (3.6)	18 (3.0)	0.529
Tumour size>50 mm, n, (%)	90 (17.3)	76 (16.2)	79 (15.0)	0.603
Preop. diagnosis PDAC, n, (%)	111 (23.5)	164 (35.0)	147 (26.9)	**<0.001**
Preop. diagnosis pNET, n, (%)	120 (25.4)	136 (29.0)	161 (29.5)	0.304
Preop. Diagnosis IPMN, n, (%)	116 (24.6)	78 (16.6)	121 (22.2)	**0.009**

Values in parentheses are percentages unless mentioned otherwise. Percentages may not add up due to rounding and missing data.

*P* values report on the statistical difference between operation years 2019, 2020 and 2021.

ASA, American Society of Anesthesiologists; BMI, body mass index; IPMN, Intraductal papillary mucinous neoplasm; PDAC, pancreatic ductal adenocarcinoma; pNET, pancreatic neuroendocrine tumor.

Statistical significance (P<0.05) values are in bold.

### Outcomes MIDP

Intra- and postoperative outcomes of MIDP are presented in Tables 3 and 4. The overall rate of intraoperative Satava grade 1 (excessive blood loss) events was 3.6% (50 patients); grade 2 (conversion or major change in operation) events 14.4% (200 patients), and grade 3 (intraoperative death) events 0% (0 patients). The main reasons for conversion were bleeding in 51 (25.5%) patients, tumor extension in 34 (17%) patients, and insufficient overview in 33 (16.5%) patients. The median operative time of MIDP was 213 min (IQR 165–274), intraoperative blood loss 100 ml (IQR 50–300), and hospital stays 7 days (IQR 5–9). The overall rate of major morbidity was 25.0% (418 patients), POPF 19.1% (318 patients), in-hospital/30-day mortality 0.6% (10 patients), readmission 15.7% (256 patients), and R0 resection 83.1% (1136 patients).

**Table 3:** Intraoperative outcome of minimally invasive distal pancreatectomy.

	Total MIDP (n=1672)	RDP (n=606)	LDP (n=1066)	*P*
Operative time, minutes, median, (IQR)	213 (165–274)	238 (180–300)	201 (155–263)	**<0.001**
Intraoperative blood loss, mL, median, (IQR)	100 (50–300)	150 (50–300)	100 (50–300)	0.202
Splenectomy, n, (%)	1037 (64.7)	365 (64.7)	672 (64.6)	0.581
Conversion, n, (%)	209 (12.5)	46 (7.6)	163 (15.3)	**<0.001**
Satava intraoperative event, n, (%)				**<0.001**
Grade 1	50 (3.6)	16 (3.4)	34 (3.7)	
Grade 2	200 (14.4)	46 (9.6)	154 (16.8)	
Grade 3	0 (0.0)	0 (0.0)	0 (0.0)	

Values in parentheses are percentages unless mentioned otherwise. Percentages may not add up due to rounding and missing data.

*P* values report on the statistical difference between RDP and LDP.

IQR, inter quartile range; Satava grade 1, excessive blood loss; Satava grade 2, conversion to laparotomy or major change in operation; Satava grade 3, intraoperative death.

Statistical significance (P<0.05) values are in bold.

**Table 4:** Postoperative outcome of minimally invasive distal pancreatectomy.

	Total MIDP (n=1672)	RDP (n=606)	LDP (n=1066)	*P*
Major morbidity, n, (%)	418 (25.0)	142 (23.4)	276 (25.9)	0.264
POPF grade B/C, n, (%)	318 (19.1)	103 (17.0)	215 (20.3)	0.098
PPH grade B/C, n, (%)	71 (4.3)	32 (5.3)	39 (3.7)	0.114
DGE grade B/C, n, (%)	19 (1.1)	9 (1.5)	10 (0.9)	0.314
Reoperation, n, (%)	69 (4.4)	28 (5.0)	41 (4.1)	0.364
Hospital stay in days, median,(IQR)	7 (5–9)	7 (5–9)	7 (5–9)	0.494
30-day readmission, n, (%)	256 (15.7)	102 (17.2)	154 (14.9)	0.224
30-day/in-hospital mortality, n, (%)	10 (0.6)	2 (0.3)	8 (0.8)	0.344
Maximum size of tumor, mm, median, (IQR)	28 (17–44)	28 (18–42)	28 (17–45)	0.892
R0 resection in PDAC, n, (%)	226 (66.1)	72 (62.1)	154 (68.1)	0.591
Total lymph nodes retrieved in PDAC, median, (IQR)	15 (9–22)	15 (8–21)	16 (9–23)	0.218

Values in parentheses are percentages unless mentioned otherwise. Percentages may not add up due to rounding and missing data.

*P* values report on the statistical difference between RDP and LDP.

DGE, delayed gastric emptying; IQR, inter quartile range; PDAC, pancreatic ductal adenocarcinoma; POPF, postoperative pancreatic fistula; PPH, post-pancreatectomy hemorrhage.

### Comparing RDP and LDP in the total cohort

No differences were observed between RDP and LDP in grade 1 and grade 3 intraoperative events. The rate of grade 2 intraoperative events was lower in RDP as compared with LDP (9.6% vs. 16.8%, *P*<0.001), as shown in Table 3, this was mainly driven by a lower conversion rate (7.6% vs. 15.3%, *P*<0.001). RDP was associated with a longer operative time (238 vs. 201 min, *P*<0.001). No significant differences were observed between RDP and LDP regarding major morbidity (23.4% vs. 25.9%, *P*=0.264) and 30-day/in-hospital mortality (0.3% vs. 0.8%, *P*=0.344). All other postoperative outcomes were also comparable between the groups (Table 4). Pathology reports from patients with PDAC revealed that the total retrieved lymph nodes (15 vs. 16, *P=*0.218), and the rate of R0 resection (62.1% vs. 68.1%, *P*=0.591) did not differ between the groups.

Risk-factors associated with intraoperative events. The multivariable analysis identified the following variables as significantly associated with a higher rate of intraoperative events: BMI greater than 25 kg/m^2^ [OR 1.534 (95% CI, 1.089–2.161), *P=*0.014], previous abdominal surgery [OR 1.549 (95% CI, 1.115–2.151), *P=*0.009], and vascular involvement [OR 1.700 (95% CI, 1.025–2.818), *P=*0.040]. Female sex [OR 0.611 (95% CI, 0.438–0.853), *P=*0.004], preoperative diagnoses pNET [OR 0.546 (95% CI, 0.361–0.825), *P=*0.004] and MCN [OR 0.497 (95% CI, 0.287–0.860), *P=*0.011], and RDP [OR 0.396 (95% CI, 0.267–0.587), *P*<.001] were significantly associated with a lower rate of intraoperative events (Supplementary Table 1, Supplemental Digital Content 2, http://links.lww.com/JS9/C137).

### Comparing RDP and LDP in high-risk groups

Three high-risk groups were identified; patients with a BMI greater than 25 kg/m^2^ (985 patients), previous abdominal surgery (472 patients), and vascular involvement (49 patients). The outcome of RDP and LDP in these groups is shown in Supplementary Table 2, Supplemental Digital Content 2, http://links.lww.com/JS9/C137. In all groups, RDP was associated with lower conversion rates and longer operative times. In the BMI greater than 25 kg/m^2^ group, RDP was associated with a higher rate of DGE (2.1% vs. 0.6%, *P=*0.042). In the previous abdominal surgery group, a lower rate of major morbidity was observed after RDP (20.0% vs. 29.5%, *P*=0.033).

### Sensitivity analysis

In the sensitivity analysis excluding patients with previous abdominal surgery, outcomes remained similar to those of the total cohort and high-risk groups. The analysis showed a higher rate of intraoperative events in LDP (14.6% vs. 8.3%, *P*=0.005), primarily due to conversion (Supplementary Table 3, Supplemental Digital Content 2, http://links.lww.com/JS9/C137).

## Discussion

This first international multicenter audit-based study in 1672 patients undergoing MIDP revealed good outcomes with mortality less than 1% and 25% major morbidity. The majority of patients were treated with LDP although the use of RDP is increasing. RDP was associated with a lower rate of grade 2 intraoperative events, mainly less conversion.

The outcomes of our study can be compared with other registries for pancreatic surgery, especially the National Surgical Quality Improvement Program (NSQIP) in North America which reported 8.6% major morbidity, 12.5% POPF grade B/C, 2.7% reoperation, 0.7% 30-day mortality, and 17.4% readmission in 1978 patients after MIDP^21^. These outcomes are largely consistent with the outcomes of this study, although the current study reported higher POPF and major morbidity rates. A clarification could be that participation in ACS-NSQIP is not mandatory and high-volume centers are more likely to participate than low-volume centers. Participating in the E-MIPS registry is not mandatory as well, however, the results of our study are based on all types of volume centers, including low-volume centers which may have contributed to higher POPF and major morbidity rates.

This study confirms the findings of two recent meta-analyses in terms of conversion and morbidity rates of RDP compared with LDP^8,9^. In both meta-analyses, conversion rates were significantly lower in RDP [OR 0.44 (0.36, 0.55)^8^ and OR 0.41 (0.33, 0.52)^9^] with comparable morbidity rates [OR 0.93 (0.76, 1.14)^8^ and OR 0.92 (0.73, 1.15)^9^. In the current study, intraoperative events were classified according to the Satava Classification to differentiate between the levels of severity. Whereas no differences were observed in grade 1 (excessive blood loss) and grade 3 (intraoperative death) events between RDP and LDP, RDP was associated with less grade 2 (conversion to laparotomy or major change in operation) events. Benefits of RDP such as the lower conversion rates have been described^8–10^ and are mainly attributed to the technical features of the robotic system providing the surgeon with more freedom of movement and better bleeding control. Although conversion in certain circumstances is necessary for a safe progression and to ensure adequate oncological clearance, literature has demonstrated that patients requiring conversion to open surgery in MIDP have worse outcomes than those whose resection is completed minimally invasive^22^.

Multivariable analysis of risk factors associated with intraoperative events in MIDP revealed that a high BMI, previous abdominal surgery, and vascular involvement were associated with more intraoperative events. However, when comparing RDP with LDP within these high-risk groups, it became evident that these risk factors were mainly related to LDP, as higher conversion rates were observed in LDP across all high-risk groups. It is important to note that the RDP cohort was relatively smaller and had fewer cases of previous abdominal surgery, which could potentially introduce a bias in the results from this group. Yet, in an unplanned sensitivity analysis excluding patients with previous abdominal surgery, LDP remained associated with a higher rate of intraoperative events.

While center volume and centers performing only RDP or LDP, as well as centers performing both RDP and LDP, did not show any significant influence on intraoperative events, the impact of surgeons’ experience could not be analyzed, as the E-MIPS registry does not collect data on operating surgeons and their caseloads in MIPS or other procedures. Surgeons who perform LDP in complex patients without sufficient experience or those who perform both RDP and LDP, failing to achieve their volume, may introduce worse intra- and postoperative outcomes^3^. Based on the findings of the present study, surgeons may consider preferring RDP over LDP in patients with a high BMI, previous abdominal surgery, or vascular tumor involvement to avoid potential conversions and its adverse consequences.

The results of this study should be interpreted in light of several limitations. First, the analyses and outcomes depend on the variables available within the E-MIPS registry^23^. For the current study, the type of DP (i.e. RAMPS, standard DP, or spleen-preserving DP), type of splenectomy (i.e. planned or unplanned), and type of spleen-preserving technique were not included in the E-MIPS registry, while these could be of interest in the comparison of RDP and LDP and could help in the future consideration for a robotic or laparoscopic approach. Meanwhile, these variables have been added to the E-MIPS registry for the benefit of future projects. Second, healthcare systems differ across Europe which might have influenced variables such as hospital stay and readmission. Third, participation in the E-MIPS registry is not mandatory. As a result, certain centers that are insecure about their outcomes or perform worse may decide to not participate which could lead to selection bias. On the other hand, 83 centers in 19 countries participated in this study, which can be considered representative of the current European practice of pancreatic surgery.

## Conclusion

In centers participating in the E-MIPS registry, MIDP is mostly performed laparoscopically, although the robotic approach is used increasingly. RDP and LDP can both be considered safe and appropriate alternatives with equivalent postoperative outcomes, but RDP was associated with lower conversion rates and longer operative time, including in high-risk subgroups. Future randomized trials should confirm these findings and assess cost differences.

## Ethical approval

The ethical board of Amsterdam UMC waived ethical approval due to the observational nature of the study and the use of pseudonymized data.

## Sources of funding

Marc G. Besselink and Mohammad Abu Hilal received grants from Intuitive Surgical and Ethicon for the design and maintenance of the E-MIPS registry.

## Author contribution

Conceptualization and design: van Bodegraven, van Ramshorst, Abu Hilal, Besselink Data acquisition: All authors.

Data curation: van Bodegraven, van Ramshorst. Formal analysis: van Bodegraven, van Ramshorst, Abu Hilal, Besselink Investigation: van Bodegraven, van Ramshorst, Abu Hilal, Besselink Methodology: van Bodegraven, van Ramshorst, Abu Hilal, Besselink Supervision: Abu Hilal, Besselink Writing original draft: van Bodegraven, van Ramshorst, Abu Hilal, Besselink Writing review and editing: All authors.

## Conflicts of interest disclosure

The authors declare no conflicts of interest.

## Research registration unique identifying number (UIN)

Name of the registry: https://www.clinicaltrials.gov/.

Unique identifying number or registration ID: NCT06160154

Hyperlink to your specific registration (must be publicly accessible and will be checked): https://clinicaltrials.gov/study/NCT06160154?term=NCT06160154&rank=1.

## Guarantor

van Bodegraven, van Ramshorst, Abu Hilal, Besselink.

## Data availability statement

The data is confidential and only available upon a reasonable request.

## Supplementary Material

## Collaborators

Marcus Holmberg, MD, PhD, Division of Surgery, Department of Clinical Science, Intervention and Technology, Karolinska Institute, Karolinska University Hospital, Stockholm, Sweden; Marie L. Capelle, MD, PhD, Department of Surgery, Erasmus MC Cancer Institute, Rotterdam, the Netherlands; Niccolò Napoli, MD, PhD, Division of General and Transplant surgery, University of Pisa, Pisa, Italy; Maxim J. L. Dewulf, MD, Department of Surgery, Maastricht University Medical Center+, University of Maastricht, Maastricht, the Netherlands; Alessandro Giani, MD, Division of Minimally Invasive Surgical Oncology, ASST Grande Ospedale Metropolitano Niguarda, Milan, Italy; Peter Holka, MD, PhD, Department of Surgery, Clinical Sciences Lund, Lund University, Skåne University Hospital, Lund, Sweden; Charles de Ponthaud, MD, PhD, Department of Hepatobiliary and Pancreatic Surgery and Liver Transplantation, AP-HP, Pitié-Salpêtrière Hospital, Sorbonne University, Paris, France; Carolina González-Abós, MD, Department of HPB and Transplant Surgery, Hospital Clinic, IDIBAPS, University of Barcelona, Spain; Marta Barros Segura, MD, PhD, Department of Hepato-Pancreato-Biliary and Transplant Surgery, Hospital Universitari Vall d’Hebron, Barcelona, Spain; Vera Hartman, MD, Department of HPB, Endocrine and Transplantation Surgery, University Hospital Antwerp, Drie Eikenstraat 655, 2650 Edegem, Belgium.
