# Supplementary material for: Minimally invasive robot-assisted and laparoscopic distal pancreatectomy in a pan-European registry a retrospective cohort study
Source: Int J Surg. 2024 Mar 18;110(6):3554–61. doi: 10.1097/JS9.0000000000001315 (PMC11175778; doi:10.1097/JS9.0000000000001315)

**SUPPLEMENTARY TABLE 1. Univariable and multivariable analyses of variables associated with intraoperative events**

|  | **Univariable analysis** | | **Multivariable analysis** | |
| --- | --- | --- | --- | --- |
| **Variables** | **OR (95% CI)** | ***P*-value** | **OR (95% CI)** | ***P*-value** |
| Age ≥ 65 years (vs. <65) | 1.158 (0.882-1.521) | 0.290 | Removed step 4 |  |
| Female sex (vs. male) | 0.551 (0.419-0.724) | <0.001 | 0.611 (0.438-0.853) | 0.004 |
| ASA classification III/IV (vs. I-II) | 1.444 (1.087-1.917) | 0.011 | Removed step 7 |  |
| BMI ≥25 kg/m^2^ | 1.514 (1.136-2.017) | 0.005 | 1.534 (1.089-2.161) | 0.014 |
| Previous abdominal surgery (yes vs. no) | 1.219 (0.912-1.629) | 0.180 | 1.549 (1.115-2.151) | 0.009 |
| Tumour size >50mm (vs. ≤ 50mm) | 1.485 (1.027-2.149) | 0.036 | 1.457 (0.951-2.231) | 0.084 |
| Preop. diagnosis PDAC (yes vs. no) | 1.866 (1.384-2.515) | <0.001 | Removed step 3 |  |
| Preop. diagnosis pNET (yes vs. no) | 0.788 (0.565-1.097) | 0.158 | 0.546 (0.361-0.825) | 0.004 |
| Preop. diagnosis IPMN (yes vs. no) | 0.804 (0.566-1.142) | 0.169 | 0.666 (0.437-1.015) | 0.058 |
| Preop. diagnosis MCN (yes vs. no) | 0.671 (0.431-1.044) | 0.077 | 0.497 (0.287-0.860) | 0.012 |
| Vascular involvement (yes vs. no) | 1.618 (1.095-2.390) | 0.016 | 1.700 (1.025-2.818) | 0.040 |
| Other organ involvement (yes vs. no) | 2.414 (1.430-4.076) | <0.001 | Removed step 5 |  |
| RDP (vs. LDP) | 0.549 (0.402-0.748) | <0.001 | 0.396 (0.267-0.587) | <0.001 |
| High volume center (vs. low volume center) | 0.974 (0.735-1.291) | 0.855 | Removed step 6 |  |
| Performing RDP or LDP (vs. performing both) | 1.078 (0.820-1.418) | 0.590 | Removed step 2 |  |
| *OR= odds ratio, BMI= body mass index, CI= confidence interval, ASA = American Society of Anaesthesiologists, PDAC pancreatic ductal adenocarcinoma, pNET= pancreatic neuroendocrine tumor, IPMN= intraductal papillary mucinous neoplasm, MCN= mucinous adenocarcinoma* | | | | |

**SUPPLEMENTARY TABLE 2. Outcome of RDP and LDP in high-risk groups (BMI of >25 kg/m2, previous abdominal surgery, and vascular involvement of the tumor)**

|  | **BMI >25 kg/m2 (n=985)** | | | **Previous abdominal surgery (n=472)** | | | **Vascular involvement (n=49)** | | |
| --- | --- | --- | --- | --- | --- | --- | --- | --- | --- |
|  | **RDP (n= 340)** | **LDP (n= 645)** | ***p*** | **RDP (n= 140)** | **LDP (n= 332)** | ***p*** | **RDP (n= 17)** | **LDP (n= 32)** | ***p*** |
| Operative time, minutes, median, (IQR) | 245 (190-300) | 208 (160-267) | **<0.001** | 240 (180-300) | 210 (158-270) | **0.001** | 314 (231-358) | 265 (200-324) | 0.164 |
| Intraoperative blood loss, mL, median, (IQR) | 100 (50-350) | 150 (100-350) | 0.071 | 100 (50-200) | 145 (50-300) | 0.121 | 200 (138-325) | 275 (100-413) | 0.820 |
| Conversion, n, (%) | 30 (8.8) | 116 (18.0) | **<0.001** | 9 (6.4) | 63 (19.0) | **0.002** | 3 (17.6) | 13 (41.9) | 0.195 |
| Satava intraoperative event, n, (%) |  |  | **<0.001** |  |  | **0.002** |  |  | 0.373 |
| Grade 1 | 9 (3.4) | 22 (3.9) |  | 4 (2.9) | 13 (4.1) |  | 0 (0.0) | 1 (4.0) |  |
| Grade 2 | 30 (11.2) | 107 (19.1) |  | 9 (6.6) | 61 (19.1) |  | 3 (16.7) | 11 (44.0) |  |
| Grade 3 | 0 (0.0) | 0 (0.0) |  | 0 (0.0) | 0 (0.0) |  | 0 (0.0) | 0 (0.0) |  |
| Major morbidity, n, (%) | 87 (25.6) | 163 (25.3) | 0.913 | 28 (20.0) | 98 (29.5) | **0.033** | 4 (23.5) | 10 (31.3) | 0.569 |
| POPF grade B/C, n, (%) | 62 (18.3) | 140 (21.8) | 0.191 | 21 (15.0) | 75 (22.8) | 0.056 | 1 (5.9) | 9 (28.1) | 0.066 |
| PPH grade B/C, n, (%) | 17 (5.0) | 19 (3.0) | 0.103 | 3 (2.1) | 12 (3.6) | 0.402 | 1 (6.3) | 0 (0.0) | 0.153 |
| DGE grade B/C, n, (%) | 7 (2.1) | 4 (0.6) | **0.042** | 0 (0.0) | 2 (0.6) | 0.357 | 1 (6.3) | 1 (3.2) | 0.626 |
| 30-day readmission, n, (%) | 63 (18.6) | 93 (14.9) | 0.136 | 24 (17.5) | 49 (14.9) | 0.486 | 0 (0.0) | 4 (13.3) | 0.126 |
| Reoperation, n, (%) | 13 (4.1) | 16 (2.6) | 0.220 | 2 (1.4) | 13 (3.9) | 0.163 | 2 (12.5) | 4 (16.0) | 0.757 |
| Hospital stay in days, median, (IQR) | 7 (5-10) | 7 (5-9) | 0.392 | 7 (6-9) | 7 (5-10) | 0.527 | 8 (7-10) | 7 (5-13) | 0.667 |
| 30-day mortality, n, (%) | 1 (0.3) | 4 (0.6) | 0.494 | 1 (0.7) | 4 (1.2) | 0.634 | 0 (0.0) | 0 (0.0) | - |
| R0 resection in PDAC, n, (%) | 47 (68.1) | 85 (73.3) | 0.654 | 34 (77.3) | 55 (72.4) | 0.834 | 5 (62.5) | 8 (61.5) | 0.965 |
| *Values in parentheses are percentages unless mentioned otherwise. Percentages may not add up due to rounding and missing data. SD= standard deviation, BMI = body mass index, ASA = American Society of Anaesthesiologists, Satava grade 1= excessive blood loss, Satava grade 2= conversion to laparotomy or major change in operation, Satava grade 3= intraoperative death, POPF= postoperative pancreatic fistula, PPH= post-pancreatectomy haemorrhage, DGE= delayed gastric emptying, IQR = inter quartile range. P-values report on the statistical difference between RDP and LDP.* | | | | | | | | | |

**SUPPLEMENTARY TABLE 3. Sensitivity analysis excluding patients with previous abdominal surgery**

|  | **No previous abdominal surgery (n=1081)** | | |
| --- | --- | --- | --- |
|  | **RDP (n= 404)** | **LDP (n= 677)** | ***p*** |
| Conversion, n, (%) | 27 (6.7) | 93 (13.8) | **<0.001** |
| Satava intraoperative event, n, (%) |  |  | **0.005** |
| Grade 1 | 12 (3.7) | 21 (3.6) |  |
| Grade 2 | 27 (8.3) | 93 (14.6) |  |
| Grade 3 | 0 (0.0) | 0 (0.0) |  |
| Major morbidity, n, (%) | 101 (25.0) | 163 (24.1) | 0.732 |
| POPF grade B/C, n, (%) | 70 (17.4) | 123 (18.3) | 0.691 |
| 30-day readmission, n, (%) | 67 (17.0) | 91 (14.0) | 0.195 |
| Reoperation, n, (%) | 25 (6.3) | 26 (3.9) | 0.081 |
| 30-day mortality, n, (%) | 1 (0.2) | 4 (0.6) | 0.421 |
| *Values in parentheses are percentages unless mentioned otherwise. Percentages may not add up due to rounding and missing data. Satava grade 1= excessive blood loss, Satava grade 2= conversion to laparotomy or major change in operation, Satava grade 3= intraoperative death, POPF= postoperative pancreatic fistula. P-values report on the statistical difference between RDP and LDP.* | | | |

**SUPPLEMENTARY FIGURE 1. Flow-chart of the study methodology**


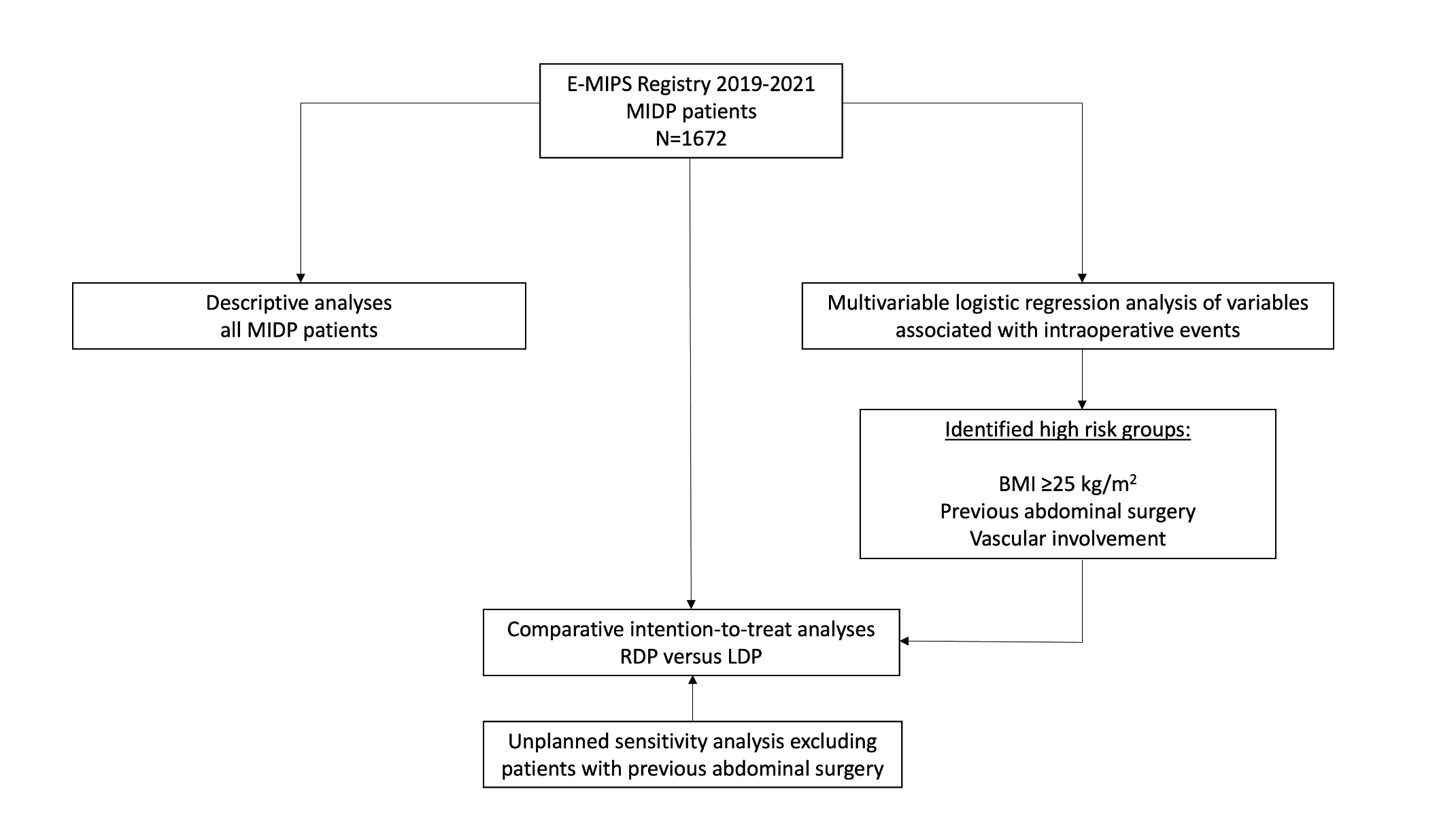


**SUPPLEMENTARY FIGURE 2. Number of MIDP procedures per center** **in 2019-2021**


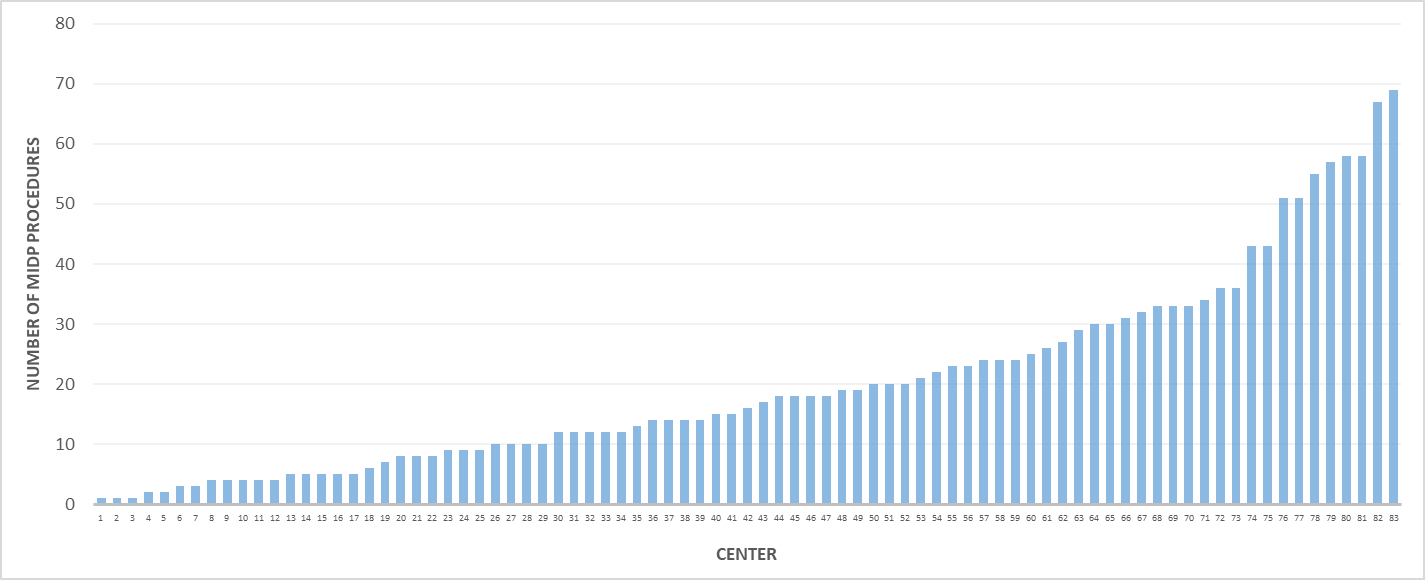

Supplement: Supplementary file 1 [file js9-110-3554-s001.docx]
